# Elucidating systemic immune responses to acute and convalescent SARS‐CoV‐2 infection in children and elderly individuals

**DOI:** 10.1002/iid3.1167

**Published:** 2024-02-01

**Authors:** Anuradha Rajamanickam, Pavan Kumar Nathella, Aishwarya Venkataraman, Padmapriyadarsini Chandrasekaran, Sasidaran Rajendraprasath, Bella D. Devaleenal, Arul Nancy Pandiarajan, Gowshika Krishnakumar, Padmasani Venkat Ramanan, Subash Babu

**Affiliations:** ^1^ Department of ICER National Institutes of Health‐National Institute for Research in Tuberculosis—International Center for Excellence in Research Chennai India; ^2^ Department of Immunology ICMR−National Institute for Research in Tuberculosis Chennai India; ^3^ Department of Clinical Research ICMR−National Institute for Research in Tuberculosis Chennai India; ^4^ Department of Paediatrics Sri Ramachandra Institute of Higher Education & Research Chennai India; ^5^ Laboratory of Parasitic Diseases National Institute of Allergy and Infectious Diseases, National Institutes of Health Bethesda Maryland USA

**Keywords:** acute phase proteins, chemokines, children, complement components, COVID‐19, cytokines, elderly, growth factors

## Abstract

**Background:**

Severe Acute Respiratory Syndrome Coronavirus‐2 (SARS‐CoV‐2), a causative pathogen of the COVID‐19 pandemic, affects all age groups. However, various studies have shown that COVID‐19 presentation and severity vary considerably with age. We, therefore, wanted to examine the differences between the immune responses of children with COVID‐19 and elderly COVID‐19 individuals.

**Methods:**

We analyzed cytokines, chemokines, growth factors, and acute phase proteins in acute and convalescent COVID‐19 children and the elderly with acute and convalescent COVID‐19.

**Results:**

We show that most of the pro‐inflammatory cytokines (interferon [IFN]γ, interleukin [IL]‐2, tumor necrosis factor‐α [TNFα], IL‐1α, IFNα, IFNβ, IL‐6, IL‐12, IL‐3, IL‐7, IL‐1Ra, IL‐13, and IL‐10), chemokines (CCL4, CCL11, CCL19, CXCL1, CXCL2, CXCL8, and CXL10), growth factors (vascular endothelial growth factor and CD40L) and acute phase proteins (C‐reactive protein, serum amyloid P, and haptoglobin) were decreased in children with acute COVID 19 as compared with elderly individuals. In contrast, children with acute COVID‐19 exhibited elevated levels of cytokines‐ IL‐1β, IL‐33, IL‐4, IL‐5, and IL‐25, growth factors—fibroblast growth factor‐2, platelet‐ derived growth factors‐BB, and transforming growth factorα as compared with elderly individuals. Similar, differences were manifest in children and elderly with convalescent COVID‐19.

**Conclusion:**

Thus, COVID‐19 children are characterized by distinct cytokine/chemokine/growth factor/acute phase protein markers that are markedly different from elderly COVID‐19 individuals.

## Graphical abstract

Children with COVID‐19 are characterized by distinct cytokine/chemokine/growth factor/acute phase protein markers that are markedly different from elderly COVID‐19 individuals. CRP, C‐reactive protein; IFN, interferon; IL, interleukin; PCA, principal component analysis; TGFα, transforming growth factorα; TNFα, tumor necrosis factorα.

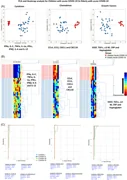

## 1 INTRODUCTION

Severe Acute Respiratory Syndrome Coronavirus‐2 (SARS‐CoV‐2) infection presents with varying severity in different age groups, with children usually having less severe disease and the elderly population presenting with severe disease.^1^, ^2^ This variation is possibly due to the disparities in the immune responses to SARS‐CoV2 among children and adults.^3^ Recently published reports demonstrated that patients aged between 30 and 79 years showed 14.8% mortality rates, while children exhibit a 1%–5% mortality.^4^, ^5^ Various studies have shown that during the infection the inflammatory response in children is less in comparison to adults.^6^, ^7^, ^8^ Comprehensive profiling of local and systemic immune responses revealed an increase in naïve lymphocytes and depletion of Natural Killer cells in children compared to adults.^9^ Similarly, children with acute coronavirus disease‐19 (COVID‐19) had a reduced breadth of anti‐SARS‐CoV2 binding and neutralizing antibodies.^10^

Previous studies have established significantly elevated levels of various immune markers, including cytokines, chemokines, growth factors, acute‐phase proteins, matrix metalloproteinases (MMPs), and indicators of microbial translocation, in children experiencing acute COVID‐19 and Multisystem Inflammatory Syndrome in Children (MIS‐C).^11^, ^12^, ^13^, ^14^ Notably, disparities in the immune system and immune senescence between children and adults may contribute to the diversity of COVID‐19 indices.^1^ Despite this, a comprehensive and systematic analysis, as well as a direct comparison of systemic immune responses in children and the elderly, are currently lacking. Therefore, the primary objective of this study is to investigate and delineate the immune response variations between children and elderly individuals at both the acute and convalescent stages of COVID‐19 infection.

## 2 MATERIALS AND METHODS

### 2.1 Study cohort

The study encompassed two distinct cohorts, with pediatric data sourced from the COVID‐19 pediatric study (CTRI/2021/01/030605). This cohort comprised 31 children, with 12 classified as acute cases and 19 as convalescent cases. The participants, aged 2 months to under 18 years, sought care at Sri Ramachandra Institute for Higher Education and Research, a tertiary hospital in Chennai, India, between August 2020 and August 2021. Inclusion criteria involved confirmed COVID‐19 infections, determined by positive reverse‐transcriptase polymerase chain reaction (RT‐PCR) results. Informed consent, and where applicable, parental or caregiver approval were obtained. Human immunodeficiency virus‐positive children were excluded. Blood samples were collected 10–21 days postpositive RT‐PCR and again at 12–16 weeks, representing acute and convalescent stages. Plasma was stored at −80°C, and subsequent analyses were conducted in batches.

Elderly individual data originated from the BCG for elderly study (NCT04475302), incorporating 149 participants aged 60–80 years. The cohort included 35 individuals with acute COVID‐19 (male *n* = 19, female *n* = 16) confirmed by PCR and 114 convalescent individuals (male *n* = 61, female *n* = 53) with SARS‐CoV‐2 immunoglobulin G (IgG) positivity. Sample collection occurred within 0–15 days of RT‐PCR confirmation and after 12–16 weeks, with recruitment spanning July to September 2020. The study adhered to cross‐sectional research reporting guidelines outlined by Strengthening the Reporting of Observational Studies in Epidemiology.

### 2.2 SARS‐CoV‐2 RT‐PCR test

In both studies, laboratories accredited by the Indian Council of Medical Research (ICMR) conducted real‐time RT‐PCR for SARS‐CoV‐2.

### 2.3 SARS‐CoV‐2 antibody assay

To assess antibodies in plasma, the iFlash™ SARS‐CoV‐2 IgG chemiluminescence antibody assay from YHLO Biotechnology Corporation, Shenzhen, China, was employed following the manufacturer's guidelines. A titer exceeding 10 AU/mL was considered indicative of a positive antibody response.

### 2.4 Multiplex assays

All tests for both studies were conducted at the National Institute for Research in Tuberculosis—International Center for Excellence in Research laboratory (NIRT‐ICER), NIRT, Chennai. The Luminex Magpix Multiplex Assay system (Bio‐Rad) was utilized to assess acute phase proteins, chemokines, and circulating plasma levels of these parameters. Acute phase proteins were quantified using the Milliplex MAP Human CVD Panel Acute Phase magnetic bead panel 3, while cytokines and chemokines were measured using the Luminex Human Magnetic Assay kit 45 Plex (R & D Systems). The lowest detection limits for all analytes are presented in Table S1.

### 2.5 Statistical analysis

Geometric means served as a measure of central tendency. Statistical differences between the elderly and children groups were analyzed using the nonparametric Mann–Whitney *U* test. Adjustments for multiple comparisons were made utilizing Holm's multiple correction method. Categorical variables are presented as numbers, medians, and proportions, while continuous variables are expressed as medians. Data analysis was carried out using GraphPad PRISM version 9 (GraphPad Software Inc.). JMP14 was employed for the analysis of dendrograms, heatmaps, and correlations.

## 3 RESULTS

### 3.1 Cytokine responses in children and elderly across the spectrum of COVID‐19 disease

**Figure 1:**
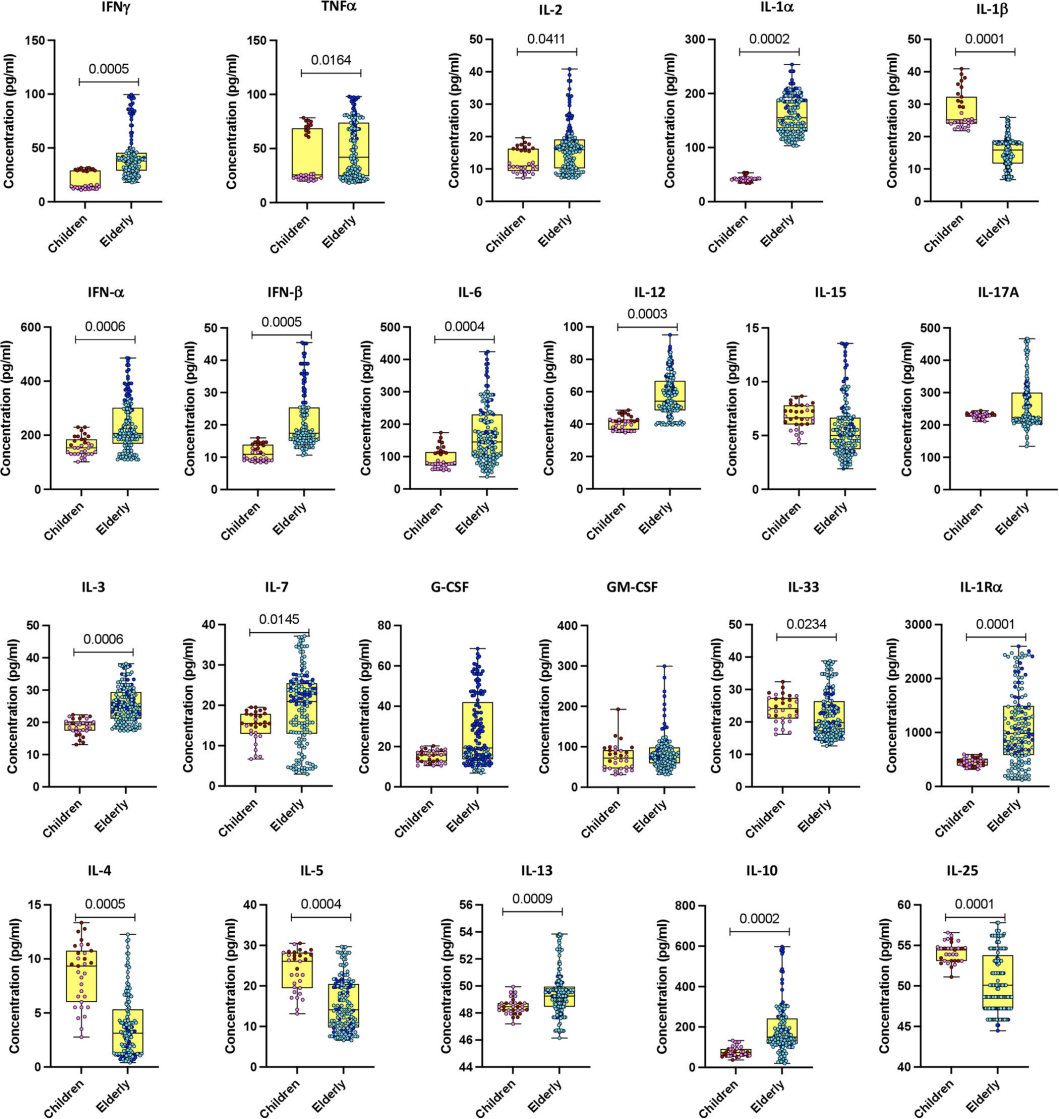
Cytokine responses in children and elderly during the acute phase of COVID‐19. Plasma levels of interferon (IFN)γ, interleukin (IL)‐2, tumor necrosis factor (TNF)α, IL‐1α, IL‐1β, IFNα, and IFNβ; IL‐6, IL‐12, IL‐15, IL‐17A, IL‐3, IL‐7, G‐CSF, and GM‐CSF, as well as IL‐4, IL‐5, IL‐13, IL‐10, IL‐25, IL‐33, and IL‐1Ra were quantified in acute COVID‐19 children (*n* = 12), convalescent COVID‐19 children (*n* = 19), acute elderly COVID‐19 individuals (*n* = 35), and convalescent elderly COVID‐19 individuals (*n* = 114). In the graphical representation, the maroon color corresponds to acute COVID‐19 children, pink represents convalescent COVID‐19 children, dark blue denotes acute elderly COVID‐19 individuals, and light blue signifies convalescent elderly COVID‐19 individuals. The data are presented as boxes and whiskers, with each circle representing an individual. Statistical significance (*p* values) was determined using the Mann–Whitney *U* test with Holm's analysis for multiple comparisons. G‐CSF, granulocyte‐colony stimulating factor; GM‐CSF, granulocyte macrophage‐colony stimulating factor.

The plasma levels of an array of cytokines (Type 1—interferon [IFN]γ, interleukin [IL]‐2, and tumor necrosis factor [TNF]α, Type 2—IL‐4, IL‐5, IL‐13, IFNs—IFNα and IFNβ), regulatory cytokines—IL‐10, IL‐25, and IL‐33 and other pro‐inflammatory cytokines—IL‐1α, IL‐1β, IL‐6, IL‐12, IL‐15, IL‐17A, IL‐3, IL‐7, granulocyte ‐colony stimulating factor, granulocyte macrophage‐colony stimulating factor, IL‐1Ra) were analyzed in COVID‐19 children and elderly patients. As depicted in Figure 1, COVID‐19 children exhibited significantly decreased levels of IFNγ, IL‐2, TNF‐α, IL‐1α, IFNα, IFNβ, IL‐6, IL‐12, IL‐3, IL‐7, IL‐1Ra, IL‐13, IL‐10 and increased levels of IL‐1β, IL‐33, IL‐4, IL‐5, IL‐25 as compared with elderly individuals. Thus, COVID‐19 children and elderly individuals demonstrated an altered level of cytokine responses.

### 3.2 Chemokine responses in children and elderly across the spectrum of COVID‐19 disease

**Figure 2:**
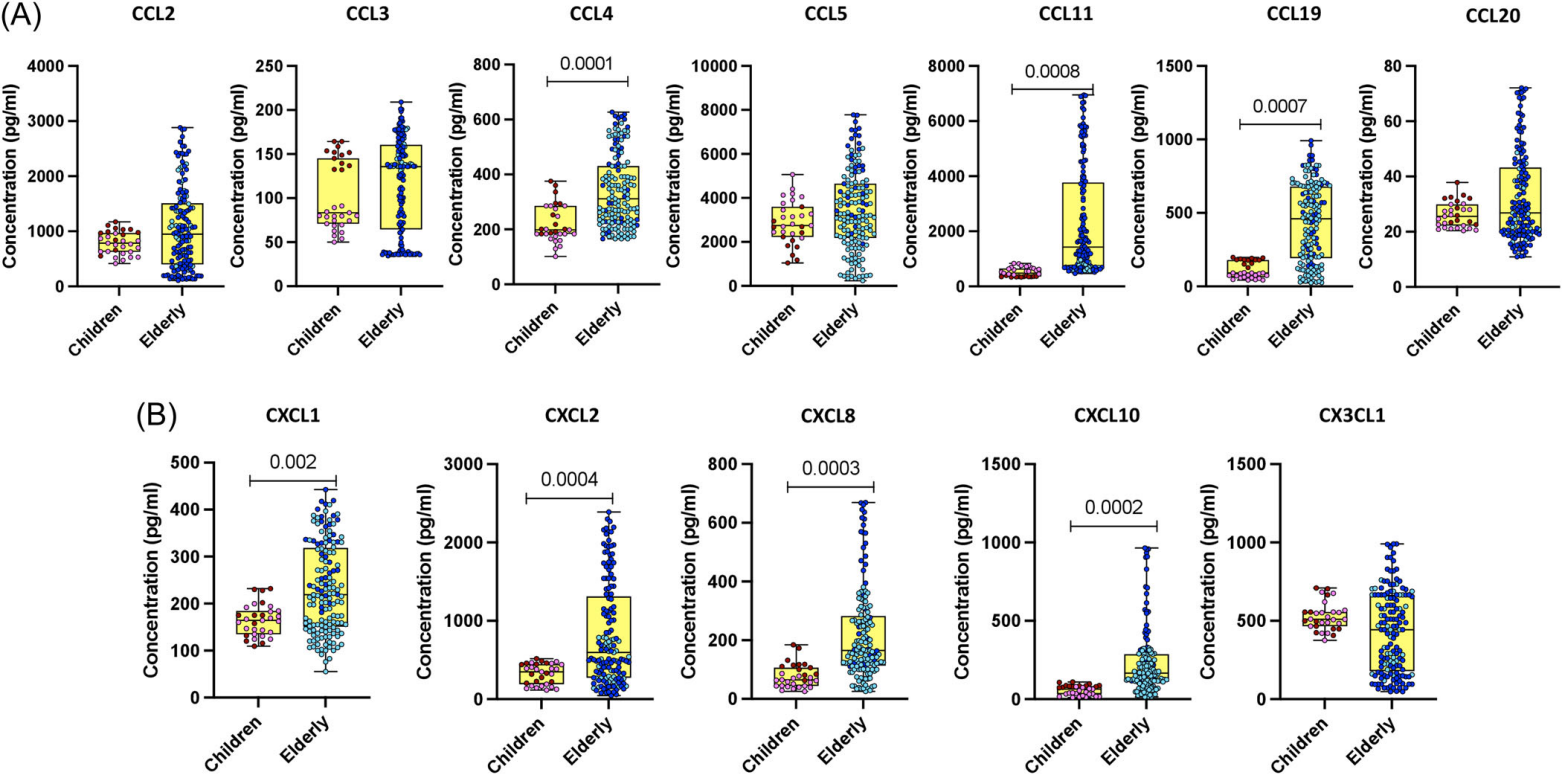
Chemokine responses in children and elderly during the acute COVID‐19 phase. (A) Plasma levels of CCL2, CCL3, CCL4, CCL5, CCL11, CCL19, and CCL20, as well as (B) CXCL1, CXCL2, CXCL8, CXCL10, and CX3CL1 were assessed in acute COVID‐19 children (*n* = 12), convalescent COVID‐19 children (*n* = 19), acute elderly COVID‐19 individuals (*n* = 35), and convalescent elderly COVID‐19 individuals (*n* = 114). In the graphical representation, the maroon color corresponds to acute COVID‐19 children, pink represents convalescent COVID‐19 children, dark blue denotes acute elderly COVID‐19 individuals, and light blue signifies convalescent elderly COVID‐19 individuals. The data are presented as boxes and whiskers, with each circle representing an individual. Statistical significance (*p* values) was determined using the Mann–Whitney *U* test with Holm's analysis for multiple comparisons.

We determined the plasma levels of an array of chemokines (CC chemokines—CCL2, CCL3, CCL4, CCL5, CCL11, CCL19, and CCL20; CXCL chemokine—CXCL1, CXCL2, CXCL8, CXCL10, and CX3CL1) in children and elderly COVID‐19 individuals. As depicted in Figure 2, COVID‐19 children exhibited significantly lower levels of CCL4, CCL11, CCL19, CXCL1, CXCL2, CXCL8, and CXL10 as compared with elderly individuals. Thus, COVID‐19 children are linked with decreased levels of chemokine responses.

### 3.3 Growth factors responses in children and elderly across the spectrum of COVID‐19 disease

**Figure 3:**
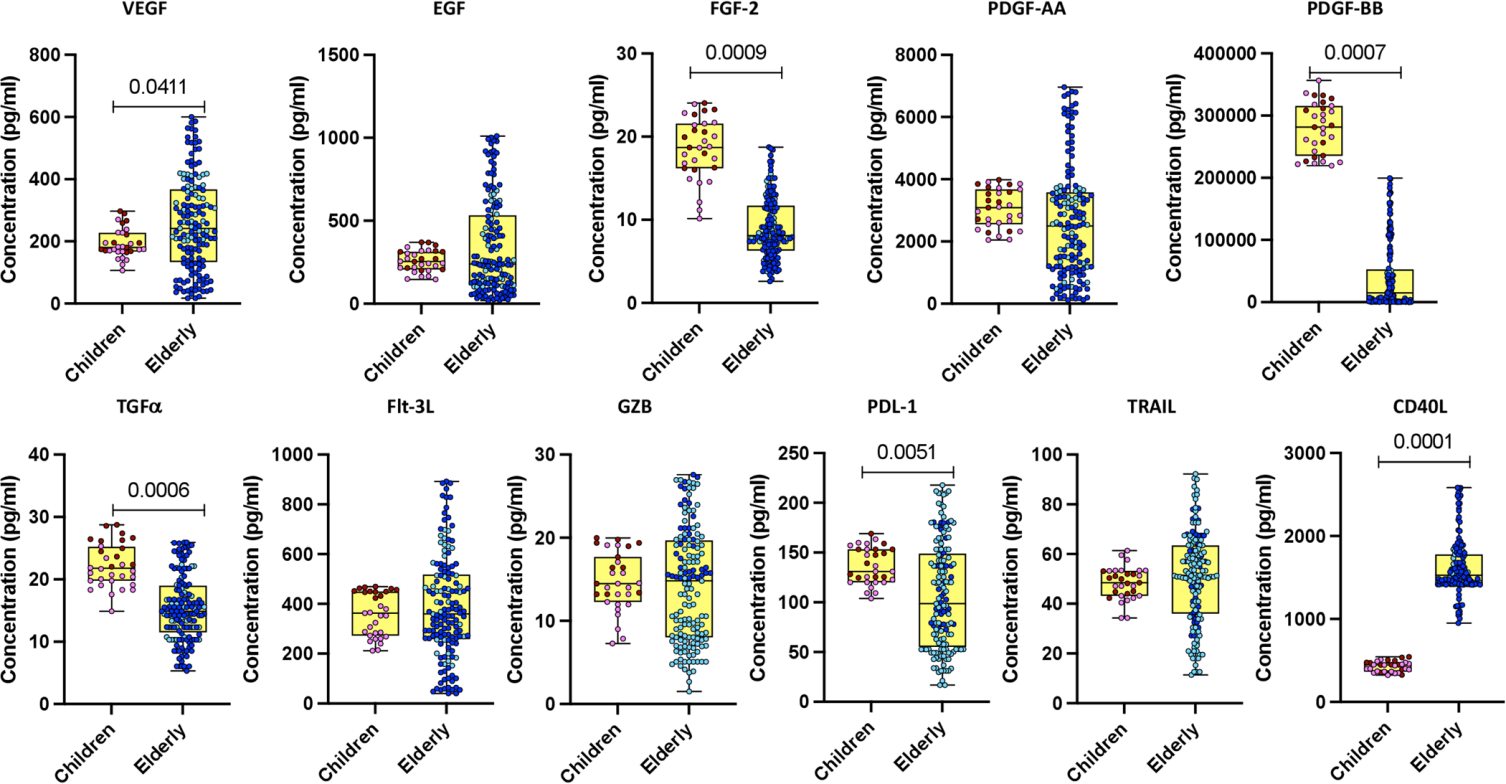
Growth factor responses in children and elderly during acute COVID‐19 phase. Plasma levels of growth factors were assessed by multiplex enzyme‐linked immunosorbent assay in acute COVID‐19 children (*n* = 12), convalescent COVID‐19 children (*n* = 19), acute elderly COVID‐19 individuals (*n* = 35), and convalescent elderly COVID‐19 individuals (*n* = 114). In the graphical representation, the maroon color corresponds to acute COVID‐19 children, pink represents convalescent COVID‐19 children, dark blue denotes acute elderly COVID‐19 individuals, and light blue signifies convalescent elderly COVID‐19 individuals. The data are depicted as scatter plots, with each circle representing an individual. Statistical significance (*p* values) was determined using the Mann–Whitney *U* test with Holm's analysis for multiple comparisons.

We determined the plasma levels of various growth factors (vascular endothelial growth factor [VEGF], EGF, fibroblast growth factor‐2 [FGF‐2], platelet‐ derived growth factors‐AA [PDGF‐AA], platelet‐derived growth factor‐BB [PDGF‐BB], transforming growth factor [TGF]α, Flt‐3L, GZB, PDL‐1, TRAIL, and CD40L) in children and elderly COVID‐19 individuals. As illustrated in Figure 3, COVID‐19 children exhibited significantly decreased levels of VEGF and CD40L as compared with elderly individuals. In contrast, COVID‐19 children exhibited increased levels of FGF‐2, PDGF‐BB, TGFα, and PDL‐1 as compared with elderly individuals. Thus, COVID‐19 children are associated with altered levels of growth factors.

### 3.4 Acute phase responses in children and elderly across the spectrum of COVID‐19 disease

**Figure 4:**
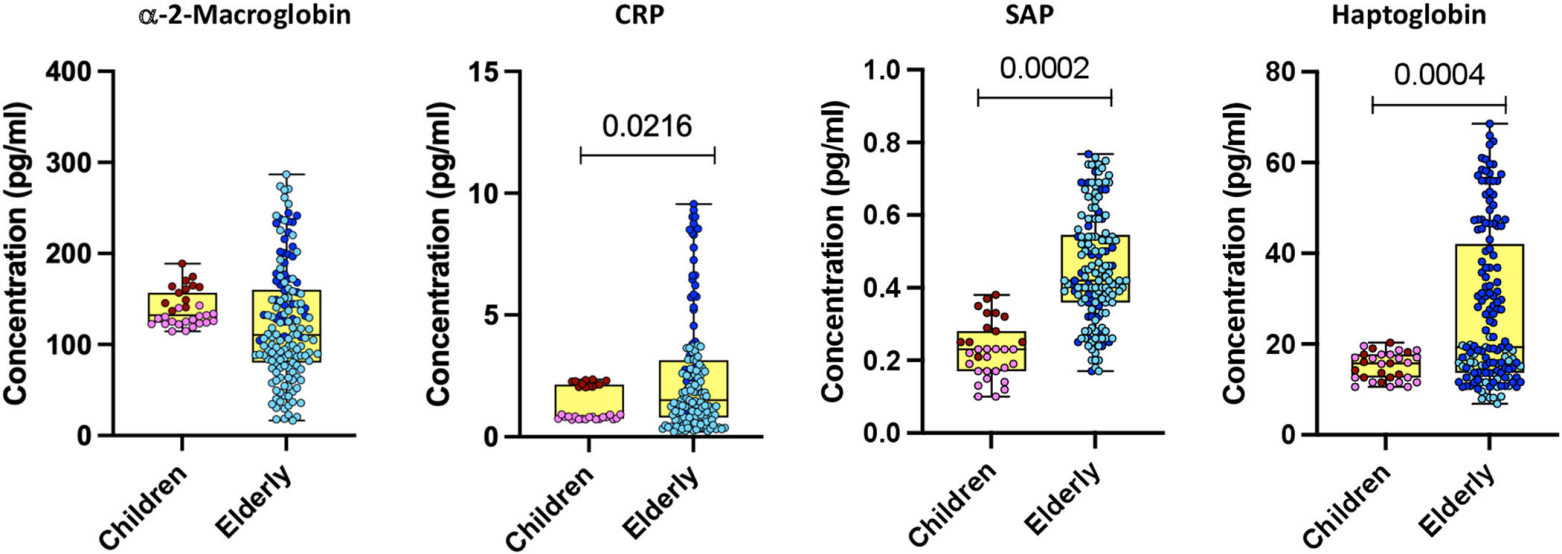
Acute phase responses in children and elderly during acute COVID‐19 phase. Plasma levels of C‐reactive protein (CRP), α‐2 macroglobulin, haptoglobin, and serum amyloid P (SAP) were quantified in acute COVID‐19 children (*n* = 12), convalescent COVID‐19 children (*n* = 19), acute elderly COVID‐19 individuals (*n* = 35), and convalescent elderly COVID‐19 individuals (*n* = 114). In the graphical representation, the maroon color corresponds to acute COVID‐19 children, pink represents convalescent COVID‐19 children, dark blue denotes acute elderly COVID‐19 individuals, and light blue signifies convalescent elderly COVID‐19 individuals. Statistical significance (*p* values) was determined using the Mann–Whitney *U* test with Holm's multiple correction analysis for multiple comparisons.

We determined the levels of acute‐phase proteins (α‐2‐macroglobulin, C‐reactive protein  [CRP], serum amyloid P [SAP], and haptoglobin) in children and elderly COVID‐19 individuals. As illustrated in Figure 4, COVID‐19 children exhibited significantly decreased levels of CRP and SAP as compared with elderly individuals. Thus, COVID‐19 children individuals are associated with heightened levels of acute‐phase proteins.

### 3.5 Plasma cytokines, chemokines, growth factors, and acute‐phase protein markers are different between children and elderly COVID‐19 individuals during the acute phase of infection

**Figure 5:**
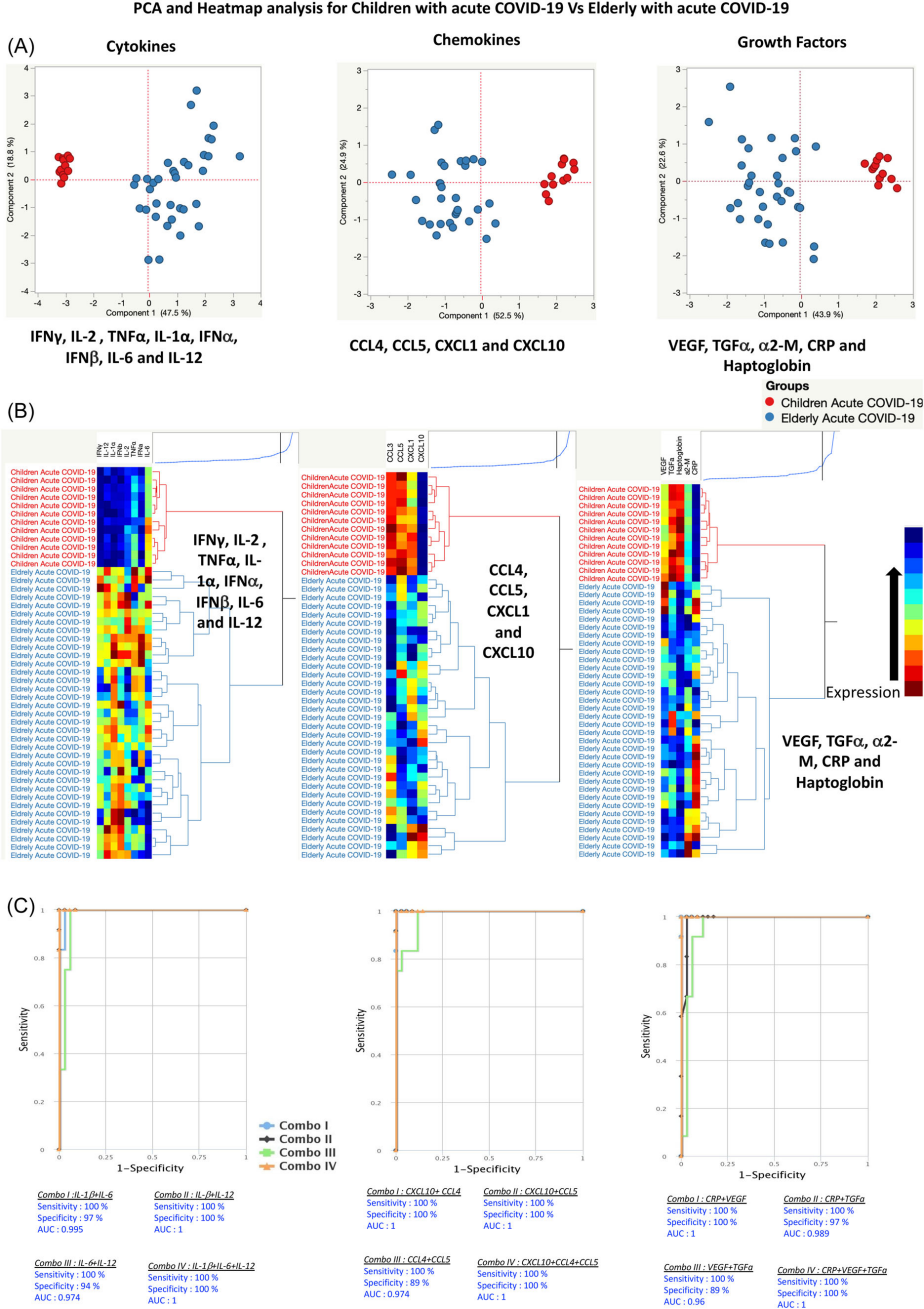
Plasma cytokines, chemokines, growth factors, and acute‐phase protein markers are different between children and elderly acute COVID‐19 individuals. (A) Principal component analysis (PCA) plot computing normalized cytokines levels after excluding those factors with commonalities as low as 0.5 we used levels of (i) cytokines parameters like interferon (IFN)γ, interleukin (IL)‐2, tumor necrosis factor (TNF)α, IL‐1α, IFNα, IFNβ, IL‐6, and IL‐12 with a combination of two different experimental groups children with acute (colored in red) versus elderly with acute COVID‐19 (colored in blue). The PCA shows the two principal components of variation for cytokines, accounting for 18.8% (*x‐*axis) and 47.5% (*y‐*axis). (ii) Normalized chemokines levels after excluding the factors with commonalities as low as 0.5, we used levels of chemokines of CCL4, CCL5, CXCL1, and CXCL10 with a combination of two different experimental groups of children with acute (colored in red) versus elderly with acute COVID‐19 (colored in blue). The PCA shows the two principal components of variation of chemokines, accounting for 24.9% (*x‐*axis) and 52.5% (*y‐*axis). (iii) Normalized growth factors and acute‐phase proteins levels after excluding the factors with commonalities as low as 0.5 we used levels of growth factors and acute‐phase proteins of VEGF, transforming growth factorα, α‐2‐M, C‐reactive protein (CRP), and haptoglobin with a combination of two different experimental groups children with acute COVID‐19 (colored in red) versus elderly with acute COVID‐19 (colored in blue). The PCA shows the two principal components of variation, accounting for 22.6% (*x‐*axis) and 43.9% (*y‐*axis). (B) (i) Cytokines, (ii) chemokines, (iii) growth factors, and acute‐phase proteins are illustrated according to the score denoted in the color‐scale bar. Associated horizontal dendrograms denote the patient's clustering, standing out clusters containing children with acute COVID‐19 (colored in red) and the elderly with acute COVID‐19 (colored in blue). On the color scale, blue color indicates higher expression and brown color indicates lower expression. (C) A plasma signature of two or three cytokines, chemokines, growth factors, and acute‐phase proteins is a precise biomarker discriminating children with acute COVID‐19 from the elderly with acute COVID‐19. CombiROC model analysis shows the cytokines that exhibited the highest accuracy in discriminating children with acute COVID‐19 from the elderly with acute COVID‐19. ROC curves for comparing multiple markers and their combinations between (i) cytokines, (ii) chemokines, (iii) growth factors, and acute‐phase proteins. ROC, receiver operating characteristic; VEGF, vascular endothelial growth factor.

Our analysis included a comprehensive range of plasma markers, namely cytokines (IFNγ, IL‐2, TNF‐α, IL‐1α, IFNα, IFNβ, IL‐6, and IL‐12), chemokines (CCL3, CCL5, CXCL1, and CXCL10), as well as growth factors and acute‐phase proteins (VEGF, TGFα, α‐2‐M, CRP, and haptoglobin) for principal component analysis (PCA). Figure 5A illustrates the effectiveness of these markers in distinguishing between acute COVID‐19 in children and geriatric COVID‐19, as demonstrated by PCA. Furthermore, to provide a comprehensive view, we conducted a hierarchical clustering analysis using data sets that incorporated levels of growth factors, cytokines, chemokines, and acute‐phase proteins from both groups.

Figure 5B presents a heatmap and dendrogram showcasing cytokines, chemokines, growth factors, and acute‐phase proteins. The dendrogram, generated using Ward's supervised clustering method and index, effectively discriminates between children with acute COVID‐19 and the elderly with acute COVID‐19, forming distinct and well‐defined clusters. This emphasizes our demonstration that a biomarker signature composed of cytokines, chemokines, growth factors, and acute‐phase proteins exhibits remarkable discriminatory performance in distinguishing between children with acute COVID‐19 and the elderly with acute COVID‐19.

### 3.6 Plasma cytokines, chemokines, growth factors, and acute‐phase protein markers are different between children and elderly COVID‐19 individuals during the convalescent phase of infection

Subsequently, we conducted PCA encompassing plasma cytokines (IFNγ, IL‐2, TNF‐α, IL‐1α, IFNα, IFNβ, IL‐6, and IL‐12), chemokines (CCL3, CCL5, CXCL1, and CXCL10), as well as growth factors and acute‐phase proteins (VEGF, TGFα, α‐2‐M, CRP, and haptoglobin). As depicted in Figure S1, the PCA vividly highlights the discriminatory capacity of these markers (cytokines, acute‐phase proteins, and growth factors) in distinguishing convalescent COVID‐19 children from elderly convalescent COVID‐19 individuals. Furthermore, utilizing data sets containing the levels of growth factors, cytokines, chemokines, and acute‐phase proteins from both groups, we conducted a hierarchical clustering analysis. Figure S1A illustrates a heatmap and dendrogram for growth factors, cytokines, chemokines, and acute‐phase proteins. Notably, the dendrogram, created using Ward's supervised clustering method and index, effectively distinguishes between convalescent COVID‐19 elderly individuals and children. Thus, we have substantiated that a biomarker signature composed of cytokines, chemokines, growth factors, and acute‐phase proteins exhibits remarkable discriminatory performance in distinguishing convalescent COVID‐19 children from elderly individuals with convalescent COVID‐19.

## 4 DISCUSSION

It is well known that COVID‐19 presents as a milder infection in children in comparison to adults or the elderly.^1^, ^2^ However, we are yet to understand clearly why there are discrepancies in the clinical indices. Some studies have shown that it could be due to age‐dependent elements that alter the antiviral immune response.^11^ It has been well known that cytokines are crucial in immunopathology during viral infection.^12^ Cytokines, TNF‐α and IFNγ, in particular, are known to drive COVID‐19 disease severity in adults.^13^ In addition, IL‐6, IL‐1β, and IL‐12 have been consistently implicated in severe disease.^13^ We have previously demonstrated that children with COVID‐19 also show a distinct cytokine/chemokine upregulation as well as alterations in acute phase proteins, complement components and MMPs.^14^, ^15^, ^16^, ^17^, ^18^ However, in this study, we show that although most of the pro‐inflammatory cytokines, chemokines, growth factors, and acute phase proteins were elevated in children, they were significantly decreased in children compared to the elderly population. Our results are similar to the finding by Pierce et al.,^13^ where they showed decreased levels of IL‐6, TNF‐α, and IP‐10 compared to adults.

Enhanced levels of acute‐phase proteins are a key feature of COVID‐19 in both pediatric as well as adult populations.^19^ CRP has been used as a predictive and diagnostic marker in COVID‐19^20^ and MIS‐C.^21^, ^22^, ^23^ Although the hyperinflammatory response in MIS‐C, appears to be similar to that seen in severe COVID‐19 in adults, there are various immunological differences.^10^ Our study demonstrates the major differences in plasma markers between children and the elderly with COVID‐19 and adds to the existing evidence that the upregulation of cytokines, chemokines, and growth factors are different in both. This could be one of the main reasons for the differences in clinical manifestations and disease severity.

Our study also extends the findings to the stage of convalescence. We have previously shown that convalescent adults are characterized by alterations in alterations in lymphocyte counts, neutrophil counts, NL ratio, monocyte counts, memory T cell subset frequencies, common γ‐chain cytokines, frequencies of classical monocytes, intermediate, and nonclassical monocytes, the activation markers sCD14, CRP, sCD163, and stissue factor, DC subsets plasmacytoid dendritic cell, myeloid dendritic cell, and IFNα, IFNβ, IFNλ1, IFNλ2, and IFNλ3, and neutrophil granular proteins such as neutrophil elastase (NE), myeloperoxidase (MPO), and proteinase‐3, and memory B cell subsets in convalescent COVID‐19 individuals.^24^, ^25^, ^26^, ^27^, ^28^ Our current study reveals that elderly individuals are associated with an enhanced immune and inflammatory response compared to children even during convalescence suggesting that the elderly might be prone to long‐term sequelae from COVID‐19. These findings will have ramifications in terms of the severity and incidence of long COVID‐19 in the elderly compared to children. The process of the immune system gives rise to a chronic pro‐inflammatory substate known as inflammaging, marked by an imbalance between stimulatory and regulatory mediators.^29^ This phenomenon can detrimentally affect overall functionality, resulting in diminished cellular responses and contributing to chronic inflammation and long‐term illnesses in the elderly.^30^ Inflammaging has the potential to amplify the host immune response, leading to cytokine storms and tissue damage. Immunosenescence further diminishes the capacity of innate immune cells to control and process viruses, while adaptive immune cells undergo reductions, limiting their ability to combat new pathogens. Studies indicate that weak immune responses and insufficient generation of SARS‐CoV‐2 antibodies may signal prolonged infection.^31^ In elderly individuals during their convalescent phase, the persistent chronic inflammatory state and decreased effectiveness of immune cells in clearing pathogens, coupled with other chronic inflammatory co‐morbid conditions, may contribute to a heightened inflammatory condition.

While acknowledging the limitations of our study, including relatively small sample size and a lack of exploration into the functional impact of the observed alterations, as well as the omission of an investigation into antigen‐specific responses, it is essential to underscore that our study significantly contributes to advancing our comprehension of the immunological and pathophysiological aspects of COVID‐19 in both pediatric and elderly populations. The nuanced insights gained from our findings serve to enrich the existing body of knowledge, laying a foundation for further research endeavors that may delve deeper into the functional implications and antigen‐specific responses associated with COVID‐19 in these demographic groups.

## AUTHOR CONTRIBUTIONS

**Anuradha Rajamanickam**: Conceptualization; data curation; formal analysis; methodology; supervision; visualization; writing—original draft; writing—review and editing. **Pavan Kumar Nathella**: Conceptualization; data curation; formal analysis; investigation; project administration; supervision; validation. **Aishwarya Venkataraman**: Formal analysis; investigation; project administration; supervision; validation; writing—review and editing. **Padmapriyadarsini Chandrasekaran**: Conceptualization; funding acquisition; project administration; resources; visualization; writing—review and editing. **Sasidaran Rajendraprasath**: Investigation; resources; supervision. **Bella D. Devaleenal**: Investigation; methodology; resources; validation; writing—review and editing. **Arul Nancy Pandiarajan**: Investigation; methodology. **Gowshika Krishnakumar**: Investigation; resources; supervision. **Padmasani Venkat Ramanan**: Conceptualization; investigation; project administration; resources; supervision; writing—review and editing. **Subash Babu**: Conceptualization; funding acquisition; investigation; project administration; resources; software; supervision; validation; visualization; writing—review and editing.

## CONFLICT OF INTEREST STATEMENT

The authors declare no conflict of interest.

## ETHICS STATEMENT

The COVID‐19 pediatric study received approval from the Institutional Ethics Committees of the National Institute for Research in Tuberculosis (NIRT‐IEC No: 2020 039) and Sri Ramachandra Institute for Higher Education and Research (SRIHER‐IEC‐NI/20/AUG/75/61). Enrollment in the study involved obtaining informed consent or assent, as applicable, from the parents or caregivers of pediatric participants. For elderly individuals, written consent was obtained before their recruitment into the study.

## Supporting information


**S. Figure** 1.

**S. Table. 1**.

## Data Availability

The data that supports the findings of this study are available in the supplementary material of this article.
