# Supplementary material for: Elucidating systemic immune responses to acute and convalescent SARS‐CoV‐2 infection in children and elderly individuals
Source: Immun Inflamm Dis. 2024 Feb 1;12(2):e1167. doi: 10.1002/iid3.1167 (PMC10832318; doi:10.1002/iid3.1167)
Supplement: Supplementary file 1 — S. Figure 1. [file IID3-12-e1167-s001.1]

Suppl.Figure.1

PCA and Heatmap analysis for Children with convalescent COVID-19 Vs Elderly with convalescent COVID-19

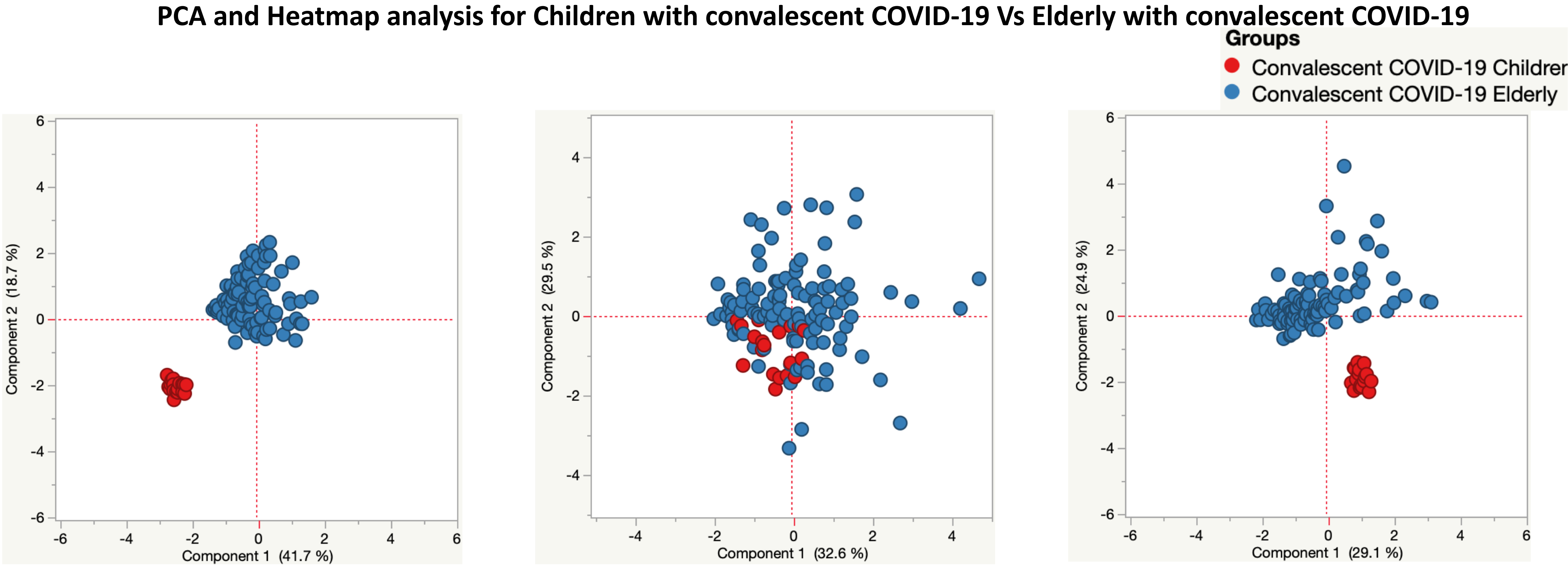

IFNγ, IL-2 , TNFα, IL-1α, IFNα, IFNβ, IL-6 and IL-12 | **CCL4, CCL5, CXCL1 and CXCL10** | **VEGF, TGFα, α2-M, CRP and Haptoglobin**

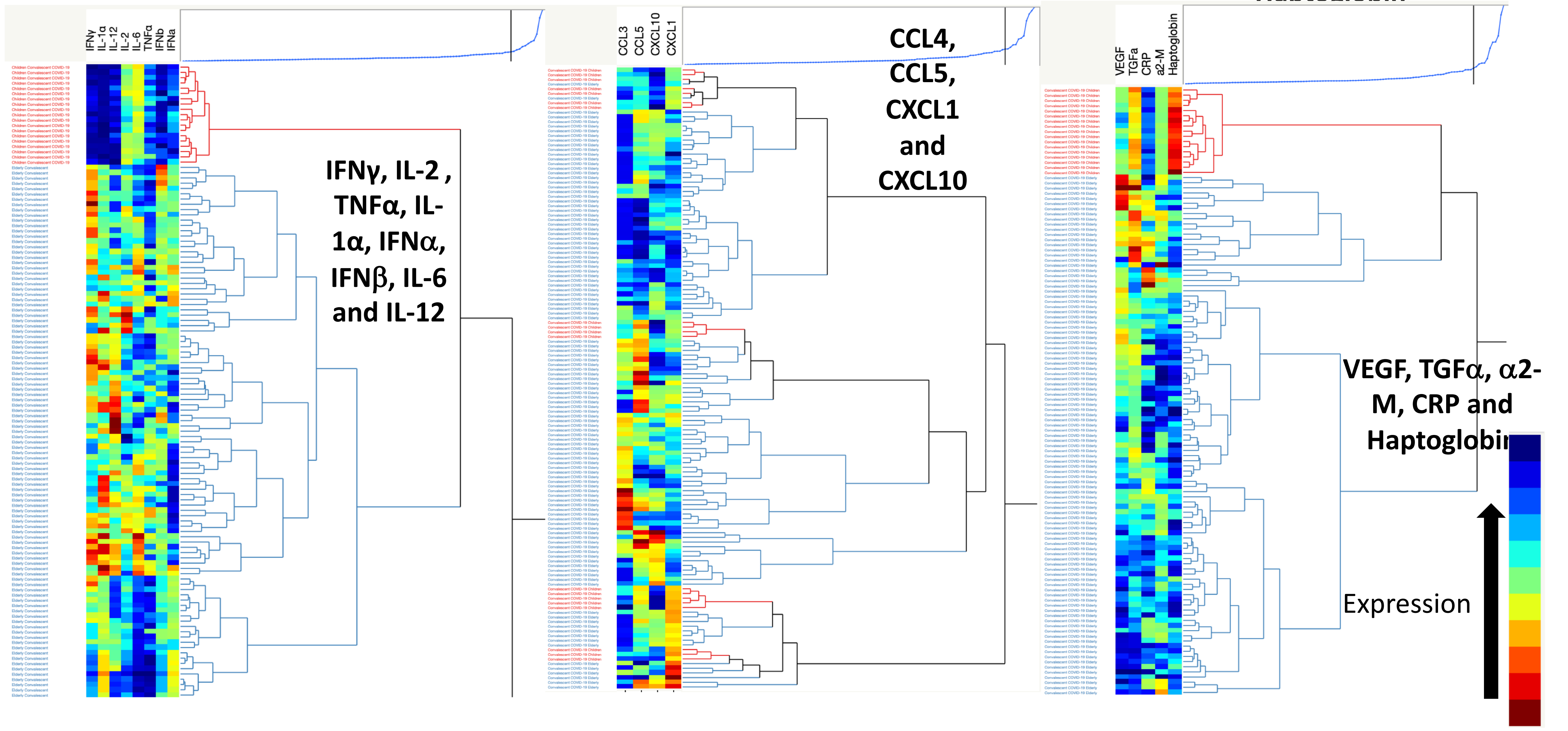

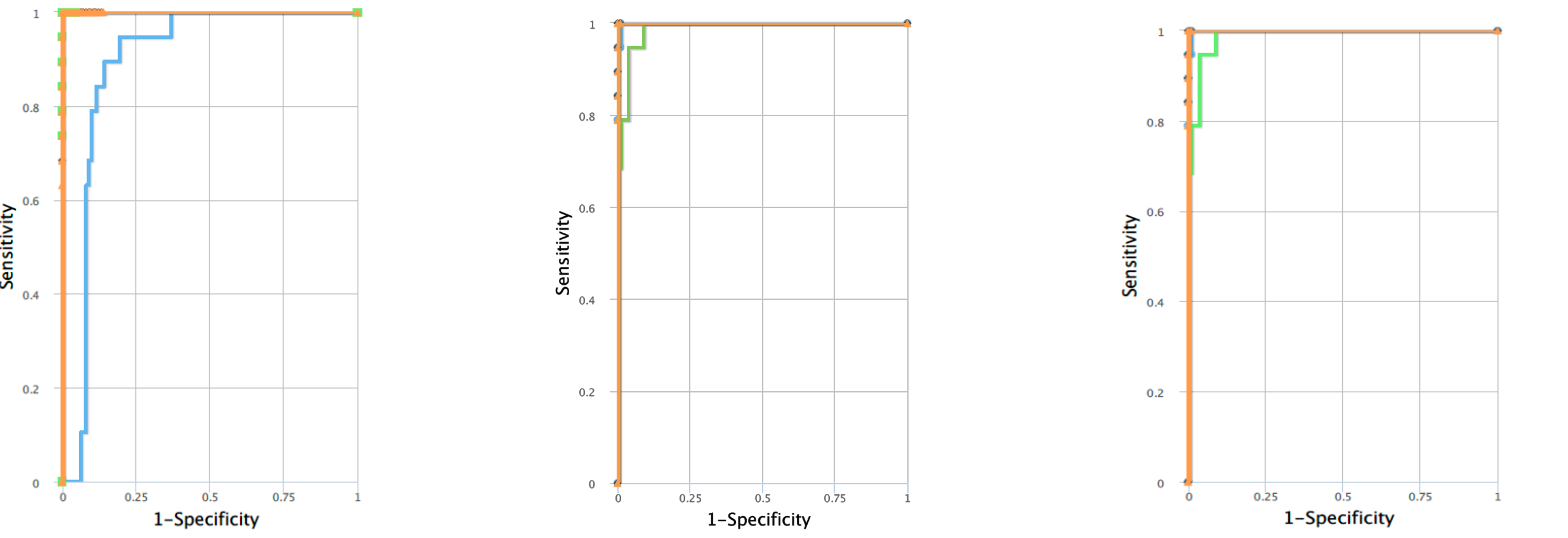

Combo I : CRP+FGF2
Sensitivity : 894 %
Specificity : 895 %
AUC : 0.86

Combo II : CRP+CD40L
Sensitivity : 100 %
Specificity : 100 %
AUC : 1

Combo I
Combo II
Combo III
Combo IV

Combo III : FGF2+CD40L
Sensitivity : 100 %
Specificity : 100 %
AUC : 1

Combo IV : CRP+FGF2+CD40L
Sensitivity : 100 %
Specificity : 100 %
AUC : 1

Combo I :IFNγ+IL-6
Sensitivity : 100 %
Specificity : 99 %
AUC : 1

Combo II : IFNγ+IL-12
Sensitivity : 100 %
Specificity : 100 %
AUC : 1

Combo III : IL-6+IL-12
Sensitivity : 100 %
Specificity : 91 %
AUC : 0.989

Combo IV : IFNγ+IL-6+IL-12
Sensitivity : 100 %
Specificity : 100 %
AUC : 1

Combo I : CCL4+ CCL11
Sensitivity : 100 %
Specificity : 100 %
AUC : 0.991

Combo II : CCL4+CXCL10
Sensitivity : 100 %
Specificity : 100 %
AUC : 1

Combo III : CCL11+CXCL10
Sensitivity : 100 %
Specificity : 91 %
AUC : 0.989

Combo IV : CCL4+CCL11+CXCL10
Sensitivity : 100 %
Specificity : 100 %
AUC : 1
