# Supplementary material for: Elucidating systemic immune responses to acute and convalescent SARS‐CoV‐2 infection in children and elderly individuals
Source: Immun Inflamm Dis. 2024 Feb 1;12(2):e1167. doi: 10.1002/iid3.1167 (PMC10832318; doi:10.1002/iid3.1167)
Supplement: Supplementary file 2 — S. Table. 1. [file IID3-12-e1167-s002.1]

| **S.Table.1. Analytes and their detection limit** | | |
| --- | --- | --- |
| **S.No** | **Analyte** | **Lowest detection limit** |
| 1 | alpha-2 macroglobulin | 0.49 ng/mL |
| 2 | C-reactive protein (CRP) | 0.05 ng/mL |
| 3 | Haptoglobin | 0.06 ng/mL |
| 4 | Serum Amyloid A-1(SAA-1) | 0.06 ng/mL |
| 5 | IFNγ | 5.7 pg/mL |
| 6 | IL-2 | 3.6 pg/mL |
| 7 | TNFα | 12.4 pg/mL |
| 8 | IL-1α | 10.6 pg/mL |
| 9 | IL-1β | 3.5 pg/mL |
| 10 | IFNα | 3.9 pg/mL |
| 11 | IFNβ | 3.25 pg/mL |
| 12 | IL-6 | 9.0 pg/mL |
| 13 | IL-12 | 18.5 pg/mL |
| 14 | IL-15 | 2.5 pg/mL |
| 15 | IL-17A | 9 pg/mL |
| 16 | IL-3 | 17 pg/mL |
| 17 | IL-7 | 3.5 pg/mL |
| 18 | G-CSF | 8.4 pg/mL |
| 19 | GM-CSF | 18.4 pg/mL |
| 20 | IL-4 | 1.1 pg/mL |
| 21 | IL-5 | 6.2 pg/mL |
| 22 | IL-13 | 31.8 pg/mL |
| 23 | IL-10 | 32.2 pg/mL |
| 24 | IL-25 | 18.4 pg/mL |
| 25 | IL-33 | 13.8 pg/mL |
| 26 | IL-1Ra | 11.7 pg/mL |
| 27 | CCL2 | 5.9 pg/mL |
| 28 | CCL3 | 5.1 pg/mL |
| 29 | CCL4 | 103.8 pg/mL |
| 30 | CCL5 | 297 pg/mL |
| 31 | CCL11 | 21.6 pg/mL |
| 32 | CCL19 | 3.9 pg/mL |
| 33 | CCL20 | 2.4 pg/mL |
| 34 | CXCL1 | 19.1 pg/mL |
| 35 | CXCL2 | 21.1 pg/mL |
| 36 | CXCL8 | 1.4 pg/mL |
| 37 | CXCL10 | 2.6 pg/mL |
| 38 | CX3CL1 | 188 pg/mL |
| 39 | VEGF | 5.9 pg/mL |
| 40 | EGF | 8.6 pg/mL |
| 41 | FGF-2 | 8.7 pg/mL |
| 42 | PDGF-AA | 5.2 pg/mL |
| 43 | PDGF-BB | 7.31 pg/mL |
| 44 | TGFa | 8.6 pg/mL |
| 45 | Flt-3L | 22.9 pg/mL |
| 46 | Granzyme B (GZB) | 4.9 pg/mL |
| 47 | PDL-1 | 69.3 pg/mL |
| 48 | TRAIL | 22.5 pg/mL |
